# Residents’ Perceptions of Effective Features of Educational Podcasts

**DOI:** 10.5811/westjem.2020.10.49135

**Published:** 2020-12-10

**Authors:** Jeffrey C. Riddell, Lynne Robins, Jonathan Sherbino, Alisha Brown, Jonathan Ilgen

**Affiliations:** * Keck School of Medicine, University of Southern California, Department of Emergency Medicine, Los Angeles, California; † University of Washington, Department of Biomedical Informatics and Medical Education, Seattle, Washington; ‡ McMaster University, Department of Medicine, Hamilton, Ontario; § Virginia Mason Hospital and Medical Center, Department of Emergency Medicine, Seattle, Washington; ¶ University of Washington, Department of Emergency Medicine, Seattle, Washington

## Abstract

**Introduction:**

Educational podcasts are used by emergency medicine (EM) trainees to supplement clinical learning and to foster a sense of connection to broader physician communities. Yet residents report difficulties remembering what they learned from listening, and the features of podcasts that residents find most effective for learning remain poorly understood. Therefore, we sought to explore residents’ perceptions of the design features of educational podcasts that they felt most effectively promoted learning.

**Methods:**

We used a qualitative approach to explore EM trainees’ experiences with educational podcasts, focusing on design features that they found beneficial to their learning. We conducted 16 semi-structured interviews with residents from three institutions from March 2016–August 2017. Interview transcripts were analyzed line-by-line using constant comparison and organized into focused codes, conceptual categories, and then key themes.

**Results:**

The five canons of classical rhetoric provided a framework for thematically grouping the disparate features of podcasts that residents reported enhanced their learning. Specifically, they reported valuing the following: 1) Invention: clinically relevant material presented from multiple perspectives with explicit learning points; 2) Arrangement: efficient communication; 3) Style: narrative incorporating humor and storytelling; 4) Memory: repetition of key content; and 5) Delivery: short episodes with good production quality.

**Conclusion:**

This exploratory study describes features that residents perceived as effective for learning from educational podcasts and provides foundational guidance for ongoing research into the most effective ways to structure medical education podcasts.

## INTRODUCTION

Podcasts are accessible and engaging learning tools, offering broad exposure to core content and personalized learning while simultaneously fostering a sense of connection to local and national professional communities. 1 In fact, emergency medicine (EM) trainees report using educational podcasts more frequently than textbooks or journals. 2–4 Yet despite their appeal, most clinicians do not listen to podcasts in a manner that aligns with evidence-based best practices for learning. 5 Some clinicians report listening to podcasts while engaged in other activities (eg, driving, exercising), where they may struggle to maintain the attention needed to comprehend the content. 1,5,6 Others report that they stop listening to educational podcasts if they don’t feel “engaged.” 1 Given these realities of podcast use, podcast producers have been encouraged to more intentionally structure podcasts in ways that keep learners interested and engaged as they fit podcast listening into their busy lives. 1

Past work has suggested that some instructional design features of podcasts might stimulate more learning than others, 1,5,7 although how these can be implemented in practice remains poorly defined. 8, 9 Drawing from existing educational theories, several features have been recommended, including interpolating questions, 10 repetition of key points, 1 short segments, 11 interview style, 12 narrative stories, 13 casual tone, 9 and written show notes. 9 Missing from past recommendations, however, is a deeper exploration of the relationship between what the technology affords and how listeners interact with it. 14,15 Exploring residents’ perceptions of their listening experiences could garner insight into how podcasts can be more intentionally designed to align with the ways that they actually incorporate them into their daily lives. 16 Thus, the purpose of this study was to explore and describe residents’ perceptions of the features of educational podcasts that they feel most effectively promote learning.

## METHODS

This was a secondary analysis of a subset of data collected for a larger study that explored how residents use podcasts for educational purposes. 1 We conducted this secondary thematic analysis of our transcripts to provide a richer and more nuanced description of the podcast features that residents reported enhanced their learning. Thematic analysis offers a flexible research approach to both examine different participant perspectives and make sense of their rich and complex narratives in ways that are illuminating and stay true to the data. 17 Recognizing that our backgrounds and assumptions influence our analysis, 18 we intentionally built our research team to include two physicians with significant experience recording, producing, and listening to medical podcasts (JR, JS), two physicians with some experience listening to medical podcasts (AB, JI), and one collaborator with background training in anthropology and extensive experience with qualitative research methods in health professions education (LR). One of the physicians (JR) also has a background in speech communication and rhetoric.

### Setting, Population, and Sampling Strategy

Expecting that trainees’ podcast listening experiences might be impacted by their backgrounds, clinical experiences, and training contexts, we purposively sampled a heterogeneous cohort of EM residents, including different postgraduate years, genders, and institutions. Participants were recruited from one Canadian institution (McMaster University) and two American institutions (University of Washington and University of California, San Francisco). The research and ethics boards at all three institutions approved the study. We invited trainees to participate by targeted emails. All participants provided verbal informed consent at the beginning of the interview and received a $25 gift card after completing the interview.

### Procedure

The principal investigator (JR) conducted hour-long, one-on-one, semi-structured interviews with participants in person or via video conference from March 2016–August 2017. We developed the interview guide based on previous surveys and the personal experiences of our research team. 2–4 We asked open-ended questions that encouraged participants to describe their listening experiences with educational podcasts, and then used probing questions to explore the particular design features that impacted their perceived learning during these experiences. Although we adapted the interview guide iteratively as the study proceeded, 19 we made no significant changes to the questions about preferred features of podcasts (eAppendix A). Audio recordings from each interview were de-identified, transcribed, and uploaded to Dedoose (SocioCultural Research Consultants, LLC, Los Angeles, CA) for data analysis.

### Analysis

Our analysis was informed by sensitizing concepts 20 drawn from classical rhetoric. 21–23 Dating back to the ancient philosophers Aristotle and Cicero, classical rhetoric provides a framework for understanding the creation of persuasive communication. 21 The five canons of classical rhetoric (invention, arrangement, style, memory, delivery) represent an organizing taxonomy of communication processes that have recently been adapted to help understand new media through the field of digital rhetoric. 24,25

We analyzed participants’ narratives iteratively alongside data collection. Using a constant comparative process, four authors (JR, AB, JI, LR) coded transcripts line-by-line to organize the data into focused codes and key conceptual categories. 18 The entire team discussed the meaning of – and connections between – these codes in light of our research questions. Codes were sorted into themes, which were further reviewed, named, and defined in discussion with the entire research team. 17 We employed memoing, triangulation of data among researchers, and an audit trail of the analytical process to enhance the trustworthiness of our analysis. 26,27 One author (JR) then coded the final six transcripts. Finding no additional insights or counterexamples, we deemed our sample theoretically sufficient to address our study purpose. 28

## RESULTS

Sixteen EM residents (5 female, 11 male) representing postgraduate years (PGY) 1 through 4/5 participated in this study, and all had past experience listening to EM podcasts. The demographics of these participants are listed in Table 1.

Participants consistently identified several themes of podcast features that impacted their perceptions of learning. We found the five canons of classical rhetoric (invention, arrangement, style, memory, delivery) to be particularly well aligned with these themes and have thus presented our data below within this organizational framework.

### Invention

The process of discovering and refining the best argument for a specific audience constitutes the rhetorical canon of invention. In our context, participants voiced a preference for podcasts that were closely aligned with their clinical experiences. In emphasizing the importance of this *clinical relevance*, they eschewed podcasts that were “too technical” or “over my head” (4, PGY-2), opting instead for content that was directly relevant to their day-to-day practice. As one senior resident elaborated:

“It has to be clinically relevant. So there are some podcasts out there that I think get very potentially bogged down in some of the minutia. For me that’s not why I listen to podcasts…. So, it’s something that is talking about how to manage a patient, and it’s not going deep into necessarily the pathophysiology, or the pharmacology, unless it’s really relevant to the discussion…” (10, PGY-4)

Residents also expressed a preference for podcast segments in which learning points were distilled and clearly signposted. More junior residents especially valued podcasters who summarized information into what they perceived to be “spoon fed” or “high yield” (5, PGY-2) take-away points. Highlighting the importance of this *emphasis on key points*, one resident explained:

“I think those things are important for learners like me that can sometimes get lost in the details and then it’s nice to at the beginning and end be told like, ‘Oh, this is what’s important about this. Here is all the details, but remember this is what’s important about this’.” (1, PGY-1)

Additionally, residents felt that podcasts that featured *multiple voices* were effective in highlighting expert clinicians’ nuanced variations in diagnosis, management approaches, or treatments. In contrast to “...someone just lecturing you”(1, PGY-1), residents elaborated on the effectiveness of conversations between hosts that “...allow people to touch on different thoughts or different nuances…” (1, PGY-1) of clinical situations. One resident explained:

“With these you get numerous different perspectives as very few of these actually have one narrator, so you’re getting different viewpoints… you’re getting guest speakers, you get specialists in certain areas… That’s a big thing.” (7, PGY-2)

### Arrangement

Arrangement represents the process of organizing arguments for maximum impact. Our participants generally valued podcasts that provided information with *efficiency*, and expressed a preference for podcast segments to make their points directly without feeling like they have to “listen forever to try to figure out what you are trying to tell me” because they were “...not going to have the patience for that.” (12, PGY-3) One participant noted:

“An effective podcast doesn’t waste a lot of time with filler space, they focus on the key information and they distill down noise about the topic into what’s most…relevant and useful when taking care of patients… they focus. They’re to the point.” (2, PGY-2)

### Style

The process of determining how an argument is presented represents the canon of style. Residents emphasized that *how* material was presented in a podcast had substantial impact on their learning. They described how humor and sound effects helped to keep them “engaged” (3, PGY-3) and “retain the information better” (3, PGY-3). Residents perceived that they were able to maintain their attention in ways that were different than traditional textbook or didactic learning, as elaborated by one participant:

“…sometimes the acting, the interludes with jokes and music and humor; it stimulates things and keeps things interesting. And it doesn’t become just a boring lecture, it becomes an entertaining means of learning” (7, PGY-2).

Multiple residents described a preference for podcasts that were conversational and included personalized accounts of clinical cases. *Storytelling* enhanced residents’ perceived engagement with podcast content, especially when experienced emergency physicians described particularly challenging or illustrative clinical experiences. One resident noted:

“Maybe they are sharing a story tied in with the information that they need you to understand. So, it kind of ties you in emotionally as well... You can hear in their voice and then you put yourself in their shoes and I think having that ability to try and visualize the experience and go through it as well is beneficial learning” (2, PGY-2).

### Memory

The canon of memory, while traditionally related to the process of memorizing a speech, also invites consideration of creating structures that make messages easy to remember. In the context of podcasts, residents overwhelmingly valued podcasts designed with repetitive segments that emphasized key points multiple times. Recapping helped to “hit it home” (5, PGY-2), cementing take-away points that may have been missed the first (or second) time. A second-year resident elaborated:

“Yeah, so I love how on [X podcast] they just kind of repeat and repeat and repeat. They summarize and then they summarize their summary... then they say it 5 minutes later again and that repetition is good.” (5, PGY-2)

### Delivery

Delivery represents the act of conveying material to an audience. Drawing comparisons to popular non-medical audio content such as National Public Radio, 29 residents valued podcasts that attended to *technical qualities* that led to pleasant listening experiences. One resident explained, “I also have [to have] good enough production quality to make it listenable” (12, PGY-3), while others reported they would stop listening if the audio quality was “bad enough that I can’t hear what people are saying.” (12, PGY-3)

Podcast *length* also seemed to impact their perceptions of whether a listening experience was effective, and residents expressed a preference toward shorter podcasts as a means to maintain focus. Longer episodes were described as “...just too much... too much noise for me to try to filter out and I don’t have time for that…” (2, PGY-2) They did not want to listen “...to the same topic for an hour” (1, PGY-1), preferring instead content that was “...bite sized, in that you can take a topic and do it in 15 minutes.” (11, PGY-2)

## DISCUSSION

These data suggest that EM residents deliberately attend to identifiable podcast features when deciding whether their listening experiences are likely to promote effective learning. Participants in this study expressed a preference for short, well-produced podcasts in which they heard multiple perspectives on clinically relevant subjects. They gravitated toward podcasts that humanized medicine through storytelling and humor while at the same time optimizing their perceived learning through efficiency and repetition of explicit learning points.

Podcasts are an oral and audible form of communication akin to the ancient Greek and Roman speeches around which the five canons of classical rhetoric were developed. 30 As such, these well-established canons can provide important insights into communication and learning from educational podcasts. 31 Whether intentionally or accidentally, podcasters appear to draw heavily on ideas from ancient rhetoricians to create educational experiences that are distinct from traditional materials (such as written texts). 32 One example is the stylistic use of music and sound effects in podcasts, which participants reported helped them stay engaged while listening. The ability to animate core medical content with analogies, anecdotes, jingles, and jokes offers new ways of making a learning experience memorable. Whether this engagement leads to more effective learning remains uncertain, as past work suggests that “cues that arise when monitoring learning and performance are often not highly predictive of actual learning and performance.” 33 Regardless, the value residents place on podcast-based learning requires careful attention to what the technology affords and how listeners interact with it. 34

Regarding memory, it is heartening to see some alignment between residents’ judgments of learning and existing literature around effective instructional techniques. Our participants’ endorsement of repetition is encouraging and aligns with the value of repetition for learning that was described over 135 years ago. 35,36 Past work, however, would suggest that optimal spacing of repetition occurs over the course of several days, not necessarily over the several minutes of a podcast segment. 37 And while some evidence supports the value of more condensed repetition timelines, 38 future work might explore the effect of repetition on long-term knowledge gain within a focused podcast segment.

Our themes that fit within the canons of invention, memory, and delivery seem to interact when viewed through the lens of cognitive load theory. It stands to reason that short segments that are repeated and summarized may be more likely to be remembered. Working memory can only process a limited amount of information at any given time, creating a “bottleneck” for learning. 39 When the cognitive load associated with a task exceeds the learner’s working memory capacity, learning is impaired. 39,40 The “distilled down” nature of podcasts may help decrease extraneous – or redundant – information; shorter segments may require less information to be held in working memory; and repetition of key information can allow content that was missed due to overloaded working memory to be revisited at a time when the listener may no longer be overloaded. 41

Likewise, our participants’ preference for high-quality sound may also reflect an important feature that minimizes extraneous load during the learning process. There are notes of caution to be sounded, however, about the cognitive load of podcast listeners. The varied contexts in which residents listen – often while exercising or driving 5 – may carry with them extraneous loads that decrease learning capacity. 42 While residents have some insight into their distracted listening, 1 further studies that explore how the rhetorical features of podcasts effect learning during simultaneous task performance may help guide effective listening behaviors.

While stories have long been an effective medium for ordering and storing complex human and clinical experiences, 43 we were surprised to hear the degree to which residents emphasized the importance of storytelling and a diversity of perspectives. Storytelling is a tool that can depict real-world clinical care, providing depth, context, and nuance around what colleagues *actually do* in practice. 44 Listening to engaging stories of how expert clinicians navigate challenging cases and conflicts may have an emotional bearing on the audience, causing them to develop strong affective responses of empathy and identification. 45 In addition to fostering a sense of connection to local colleagues and national communities, our resident participants also emphasized the correlation between feeling connected to their storytellers and the learning they gleaned from listening to their stories. 1

While this paper departs from existing rhetorical analysis of popular podcasts such as *Serial* *46* and *Revisionist History* *30* by examining listeners’ experiences with a broader genre, thinking about medical education podcasts as rhetorical objects can deepen our understanding and facilitate our investigation of the role of language in medical training. 47 We can begin to extend our attention beyond the mere ordering of medical facts outlined in a podcast to consider the social values and goals represented by, and reflected in, the platform. 48 Moving beyond traditional instructional design features, we can consider the ways stories stimulate the imagination, drawing listeners in and keeping their attention so that they remain ready for learning. 45 This move emphasizes what language does, not just what it says, 48 and transfers our attention from what is said (content) to what is accomplished (action). 47 This rhetorical frame might help explain why podcasts remain such a popular learning resource for trainees; like ancient Roman orators, podcasters – by accident or intention – appear to be effective rhetors, using storytelling, multiple voices, repetition, humor, and sound effects as persuasive means of affecting perceptions of learning.

## LIMITATIONS

Our study carries several limitations. Our purposive sampling of EM residents in the Western United States and Canada may not represent the preferences of trainees in other disciplines or geographies. The greater representation of males compared to females in our study, while not far from the gender representation of EM trainees on the whole, may limit transferability across learners.^49^ Further, we did not define “effectiveness” for our interviewees, opting instead to allow them to determine how they *perceived* effectiveness. Our representations of residents’ perceptions of effectiveness must be contextualized within the literature on the limitations of self-assessment and judgments of learning. 50–52 These findings should serve as a guide to future realist evaluations that examines these features to determine what works, where, and why, 53 rather than be viewed as a definitive prescription for podcast optimization.

## CONCLUSION

Emergency medicine residents deliberately attend to the rhetorical features of podcasts to decide whether their listening experiences are likely to promote effective learning. Residents feel they learn from short, well-produced podcasts in which they hear multiple perspectives on a clinically relevant subject. They gravitate toward podcasts that humanize medicine through storytelling and humor, while at the same time optimizing their perceived learning through efficiency and repetition of explicit learning points. The five canons of classical rhetoric may provide a useful framework to understand the rhetorical nature of podcasts and their influence on perceptions of learning.

## Supplementary Information



## Figures and Tables

**Table 1:** Characteristics of participants (n = 16).

Postgraduate year (PGY)	
PGY-1	2 (13%)
PGY-2	7 (44%)
PGY-3	4 (25%)
PGY-4/5	3 (19%)
Institution	
McMaster University	5 (31%)
University of California, San Francisco	5 (31%)
University of Washington	6 (38%)
Gender	
Female	5 (31%)
Male	11 (69%)
