# Supplementary material for: Residents’ Perceptions of Effective Features of Educational Podcasts
Source: West J Emerg Med. 2020 Dec 10;22(1):26–32. doi: 10.5811/westjem.2020.10.49135 (PMC7806333; doi:10.5811/westjem.2020.10.49135)
Supplement: Supplementary file 1 [file wjem-22-26-s001.docx]

**Appendix A**

Final Interview guide

Questions:

1. Tell me about the last podcast you listened to…
   1. Probe: When and where do you listen?
   2. Probe: How long do you typically listen in a given session?
   3. Do you go back and finish partially listened to podcasts?

Tell me more about why you keep coming back to it?

- 1. If you’re doing something else, what do you think about while listening?
  2. For the podcasts you listen to regularly, how do you feel if you miss a month? Why do you feel guilty? Do you feel that way about other resources?
  3. Do you consider podcast listening to be a habit? Or something that you try to use regularly? Have you made a conscious effort to make this a regular thing

1. Why have you chosen to learn content via podcasts?
   1. Probe: What do you like about them?
   2. Probe: What other educational resources do you frequently use? What is different about podcasts??
   3. What do you want to get out of the listening?
   4. How do you use what you get out of listening?
   5. How do you choose what to listen to?
   6. PGY -3/4: Has the way you choose to listen changed over course of your training?

Has what you do with the info you get changed over course of your training?

I’ve heard about podcasts and a sense of community, do podcasts connect you to anything or anyone?

- 1. Do you ever talk to other people about podcasts?
  2. we’ve heard about podcasts connecting people, some have sued the word community, do you experience that
  3. Tell me about connections between podcasts and your clinical work

1. What defines an effective podcast listening experience?
   1. Probe: What are some of your favorite podcasts? Why?
   2. Probe: If you could design a high yield podcast for residents, any way you want, how would you design the most effective podcast?
   3. Why do you typically stop listening to a podcast?
   4. How do you determine trustworthiness?
2. Is there anything we didn’t discuss or anything else you want to add about your experience with podcasts? Any big picture thoughts about podcasts in EM for you?
